# US Obesity Mortality Trends and Associated Noncommunicable Diseases Contributing Conditions Among White, Black, and Hispanic Individuals by Age from 1999 to 2017

**DOI:** 10.1007/s42399-021-00850-2

**Published:** 2021-04-05

**Authors:** Federico Gerardo de Cosio, Beatriz Diaz-Apodaca, Amanda Baker, Miriam Patricia Cifuentes, Hector Ojeda-Casares, Daniel Constandce, Francisco Becerra

**Affiliations:** 1 College of Health Science, University of Texas at El Paso, 1851 Wiggins Rd., El Paso, 79968 Texas USA; 2 Research and Graduate Studies, Universidad Autonoma de Ciudad Juarez, Ciudad Juárez, Mexico; 3 Ministry of Social Development and Family of Chile, Santiago, Chile; 4 Ministerio de Salud y Protección Social, Bogotá, Colombia; 5 Pan American Health Organization Venezuela, Caracas, Venezuela; 6 Universidad Autonoma de Ciudad Juarez, Ciudad Juárez, Mexico; 7 Public Health Developments Organization, Washington, DC USA

**Keywords:** Obesity, Cardiovascular disease, Diabetes, Chronic respiratory disease, Hazard ratios (HRs)

## Abstract

This study aims to assess the effect of obesity as an underlying cause of death in association with four main noncommunicable diseases (NCDs) as contributing causes of mortality on the age of death in White, Black, and Hispanic individuals in the USA. To estimate mortality hazard ratios, we ran a Cox regression on the US National Center for Health Statistics mortality integrated datasets from 1999 to 2017, which included almost 48 million cases. The variable in the model was the age of death in years as a proxy for time to death. The cause-of-death variable allowed for the derivation of predictor variables of obesity and the four main NCDs. The overall highest obesity mortality HR when associated with NCD contributing conditions for the year 1999–2017 was diabetes (2.15; 95% CI: 2.11–2.18), while Whites had the highest HR (2.46; 95% CI: 2.41–2.51) when compared with Black (1.32; 95% CI: 1.27–1.38) and Hispanics (1.25; 95% CI: 1.18–1.33). Hispanics had lower mortality HR for CVD (1.21; 95% CI: 1.15–1.27) and diabetes (1.25; 95% CI: 1.18–1.33) of the three studied groups. The obesity death mean was 57.3 years for all groups. People who die from obesity are, on average, 15.4 years younger than those without obesity. Although Hispanics in the USA have a higher prevalence of diabetes and cardiovascular disease (CVD), they also have the lowest mortality HR for obesity as an underlying cause of death when associated with CVD and cancer. While there is no obvious solution for obesity and its complications, continued efforts to address obesity are needed.

## Introduction

The prevalence of overweight and obesity has nearly tripled since 1975 worldwide, with obesity emerging as one of the most important chronic diseases and a major burden for society [1]. It is estimated that obesity was responsible for 4.7 million deaths and 148 million disability-adjusted life-years in 2017 [2]. The prevalence of obesity in the United States of America (USA) from 2015 to 2016 was 39.8%, affecting 93.3 million US adults and making it the highest obesity prevalence in OECD countries [3, 4]. There is a higher prevalence of obesity among women than men, as well as a higher prevalence among non-Hispanic Black individuals (46.8%) and Hispanic individuals (47.0%) than non-Hispanic White individuals (38.0%) [4]. It is well-documented that obesity is associated with an increased risk of disease, although it is identified as an underlying cause of death in less than 20% of deaths in the USA [5].

It is increasingly apparent that obesity and premature mortality are directly related. It is well-known that overweight and obesity are associated with increased risk of mortality from (CVD), type 2 diabetes, and certain cancers, among other noncommunicable diseases (NCDs) [6], although this association varies between populations and causes of death [7, 8]. The risk of mortality increases directly in relation to the number of years lived with obesity [8], up to 64% compared to 19% in persons with normal weight, with no difference by race or sex [9, 10].

Obesity was first included in the International Classification of Diseases Sixth Revision (ICD-6) in 1948 [11] with the code E66, which was initially used either as a cause or as a contributing cause of death rather than a disease in its own right. In 2008, a panel of experts convened by The Obesity Society concluded that considering obesity as a disease had positive consequences [12], and in 2013, the American Medical Association voted to declare obesity a disease [13, 14].

Despite the fact that the link between obesity and mortality has been well-established since the 1980s [15], the recognition of obesity’s contribution to death and of obesity-associated conditions is limited on death certificates [16]. Currently, there are very few studies using mortality vital statistics databases to analyze obesity-related mortality [5], including its associated conditions with the underlying cause of death. A review of the mortality databases of the Pan American Health Organization (PAHO) for the region of the Americas [17] found that the use of the E66 code for certifying obesity as the underlying cause of death increased from 4,050 in 1999 to 12,087 in 2015, with the USA accounting for 64.4% and 61.1% of these deaths, respectively. The underreporting of obesity as a cause or contributing factor to death can lead to underestimations in regard to the effect of obesity on mortality [18–20].

Although different studies have found that excess weight is a risk factor for diseases and mortality, other studies have documented that individuals with excess weight and NCDs live longer than individuals with a normal weight, giving rise to the “obesity paradox.” Overweight or grade/class 1 obesity is associated with significantly lower all-cause mortality [21, 22], but grades/classes 2 and 3 obesity are associated with significantly higher all-cause mortality [23, 24]. Moreover, other studies report that the relationship between body mass index (BMI) and mortality exhibits a J-shaped [3] or a U-shaped curve [21].

The reporting of race and ethnicity on death certificates accuracy is high for White and Black populations, and for Hispanics “is almost as good as that of white and black populations” [25]. In the USA, the positive predictive value of ICD 10 codes for obesity administrative diagnosis is above 92%, with a higher likelihood of using the E66 code with grade/class 1 or 3 [26].

The objective of this study was to assess the effects of obesity as an underlying cause of death in association with four main NCDs—cardiovascular disease, diabetes, cancer, and chronic respiratory disease—as contributing causes in relation to the age of death in White, Black, and Hispanic individuals in the USA.

## Methods

We integrated all the mortality datasets from 1999 to 2017 from the US National Center for Health Statistics, obtaining data from almost 48 million individuals registered [27], including people of all ages. Integration included matching all equivalent variables in the death certificate records according to the corresponding data dictionaries.

During processing, we generated indicator variables to identify the cases with any of the selected health conditions as the cause of death originally coded according to the International Classification of Diseases, Tenth Revision (ICD-10). We adopted the codes defined by the World Health Organization [28]: obesity E66; circulatory diseases I00–I99; cancer C00–C97; diabetes E10–E14; and chronic lower respiratory diseases J40–J47. For this analysis, the data on the underlying cause of mortality were disaggregated into deaths that had the code E66 (obesity) to be compared with all other codes that contributed to the main cause of death. Estimations were made for the annual and total percentage of deaths with obesity as an underlying cause of death and its contributing NCDs as cause of death in the selected studied period. All race/ethnic groups include all deaths.

We used population data by age, sex, and race (White and Black) and ethnicity (Hispanic origin), as defined by the US census as they represent more than 95% of all obesity deaths) from the Centers for Disease Control and Prevention (CDC) WONDER [29] as denominators for descriptive background analysis based on mortality rates. We included in the analyses sex, race, and ethnicity (Hispanic origin group) as covariates that could also have effects on the results. We also defined age strata of 0–14, 15–29, 30–49, 50–69, 70–84, and older than 84 years.

A Cox regression was used to estimate mortality hazard ratios (HRs) with 95% confidence intervals (95% CIs). For the Cox regression, age was used as the time to death in simple years for all cases. Obesity, according to the described coding, was the factor variable. There were no censored cases, because of the type of data. To examine the single and simultaneous effect of factors besides obesity, we included as regressors (predictor variables) each of the four NCDs listed above, age, race/ethnicity, and sex, available in the dataset.

Descriptive analyses framed our understanding of obesity as an underlying cause of death. Trends of death rates presented an evolution and magnitude, while frequencies according to sex, age groups, and ethnicity showed differential distribution.

By examining death rates, we addressed the risk of death associated with obesity and NCDs in the population. The average age of death and hazard ratios from Cox regressions allowed us to evaluate the relative effect of obesity on the length of life. To run all of these analyses, we used the SPSS program version 26.

The results are presented in tables as frequencies, mortality rates, and mortality hazard ratios. Figures are also included to identify tendencies in the studied period.

## Results

Between 1999 and 2017, there were 47,812,945 deaths, of which 99,388 were related to obesity as the underlying cause of death (49.9% male and 50.1% female) in all race/ethnic groups. For the purpose of this study, only 97,689 deaths met the race/ethnicity inclusion criteria (White, Black, and Hispanic); of these, 77,846 (50.2% male and 49.8% female) had at least one of the selected four NCDs associated with multiple causes of death.

During the study period, there was a 276% increase in the reporting of obesity in the death certificates, from 2061 in 1999 to 7752 in 2017 (189.6% in White, 179.9% in Black, and 361% in Hispanic individuals). Of the total cases with obesity as an underlying cause of death, 72,321 (72.8%) were accounted for by White individuals, 18,377 (18.5%) by Black individuals, and 6991 (7.1%) by Hispanic individuals (Table 1a).

**Table 1:** Obesity mortality characteristics by race/ethnicity, USA 1999–2017. *1,000,000 inhabitants

**1a. Distribution of total deaths by age group and race/ethnicity**					
**Age group**	**All Ethnic groups**	**White**	**Black**	**Hispanic**	**Other Races**
0-14	84	36	23	20	5
15-29	3585	1647	1188	589	161
30-49	27562	17137	6984	2745	696
50-69	48689	37529	7840	2685	635
70-84	16961	13924	2066	794	177
>85	2507	2048	276	158	25
Total	99388	72321	18377	6991	1699
%	100	72.77	18.49	7.03	1.71
**1b. Average age (years) by cause of death and race/ethnicity**					
**Cause of death**		**All ethnic groups**	**White**	**Black**	**Hispanic**
All Cause without obesity		72.69	74.66	64.78	63.66
Non communicable disease		74.91	76.1	69.3	70.73
Obesity		56.54	58.4	51.91	50.98
Obesity with one or more NCD		57.33	59.11	52.59	52.69
**1c. Percentage of deaths by age group**					
**Cause of death**	**Age group**	**All ethnic groups**	**White**	**Black**	**Hispanic**
All Cause	0-14	1.1	0.9	3.4	4.9
	15-29	1.8	1.5	3.9	5.8
	30-49	6.2	5.7	12.3	12.9
	50-69	22.5	22.2	32.8	27
	70-84	36.6	37.1	30.1	29.9
	>85	31.7	32.6	17.3	19.5
	Total	100	100	100	100
Non communicable disease	0-14	0.2	0.1	0.3	0.7
	15-29	0.4	0.3	0.9	1.3
	30-49	4.7	3.7	9.1	8.5
	50-69	26.4	24.4	37.4	31.2
	70-84	39.7	40.7	34.9	36.9
	>85	28.6	30.8	17.4	21.4
	Total	100	100	100	100
Obesity	0-14	0.1	0	0.1	0.3
	15-29	3.6	2.3	6.5	8.4
	30-49	27.7	23.7	38	39.3
	50-69	49	51.9	42.7	38.4
	70-84	17.1	19.3	11.2	11.4
	>85	2.5	2.8	1.5	2.3
	Total	100	100	100	100
Cardiovascular Disease	0-14	0.1	0.1	0.3	0.5
	15-29	0.3	0.2	0.9	0.9
	30-49	4.2	3.2	9.2	7.2
	50-69	20.2	17.8	32.9	26
	70-84	36.8	37.1	34.2	36.7
	>85	38.5	41.6	22.4	28.7
	Total	100	100	100	100
Diabetes	0-14	0.1	0	0.1	0.1
	15-29	0.6	0.4	1.1	0.7
	30-49	6.2	5.2	9.4	7.7
	50-69	31.4	28.8	38.4	36.1
	70-84	40.7	41.9	36.4	39.9
	>85	21.1	23.6	14.6	15.5
	Total	100	100	100	100
Cancer	0-14	0.2	0.2	0.3	1
	15-29	0.5	0.4	0.8	1.9
	30-49	5.8	4.9	9	11
	50-69	35.9	34.3	44.5	38.8
	70-84	41.9	43.4	34.9	35.7
	>85	15.7	16.8	10.5	11.5
	Total	100	100	100	100
Chronic Respiratory Disease	0-14	0.2	0.1	1.2	0.8
	15-29	0.2	0.1	1.4	0.8
	30-49	1.6	1.3	5.8	3.2
	50-69	22.1	21.4	32.5	20.4
	70-84	49.8	50.6	41.7	44.7
	>85	26.1	26.5	17.4	30
	Total	100	100	100	100
1d. Total number of deaths and mortality rates* associated with obesity and as underlying cause and selected NCD.					
**Cause of death**		**All ethnic groups**	**White**	**Black**	**Hispanic**
Cardiovascular Disease		70,312	50,720	13,823	4,612
Rate		12.2	11.02	17.74	5.15
Diabetes		18,416	13,648	3,125	1,308
Rate		3.2	2.97	4.01	1.46
Cancer		1,468	1,174	200	66
Rate		0.25	0.26	0.26	0.07
Chronic Respiratory Disease		13,837	10,822	2,109	723
Rate		2.4	2.35	2.71	0.81

*1.000.000 in habitants

**Fig. 1:**
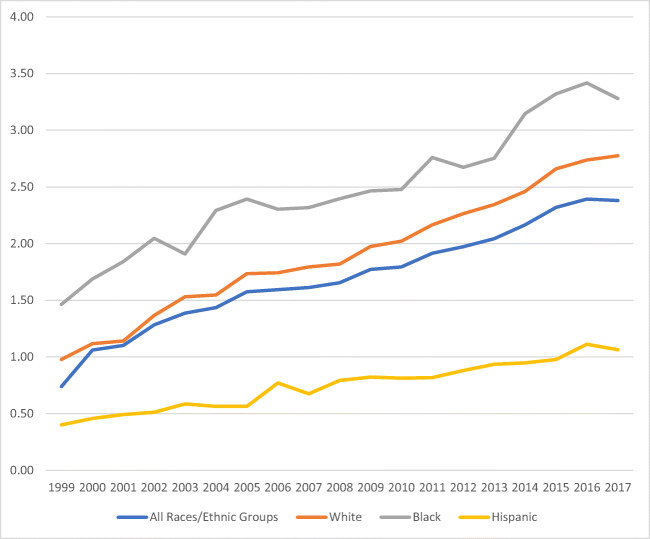
Mortality rate* associated with obesity as an underlying cause of death in all three races/ethnic groups (White, Black, and Hispanic) in the USA from 1999 to 2017. *Rate per 100,000. Source: Mortality trends constructed from the US Mortality Database 1999–2017. Population obtained from the CDC WONDER database platform

**Fig. 2:**
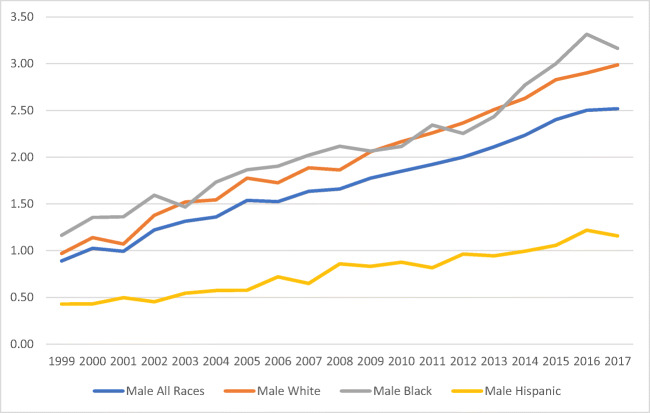
Male mortality rate* associated with obesity as an underlying cause of death in all three races/ethnic groups (White, Black, and Hispanic) in the USA from 1999 to 2017. *Rate per 100,000. Source: Mortality trends constructed from the US Mortality Database 1999–2017. Population obtained from the CDC WONDER database platform

**Fig. 3:**
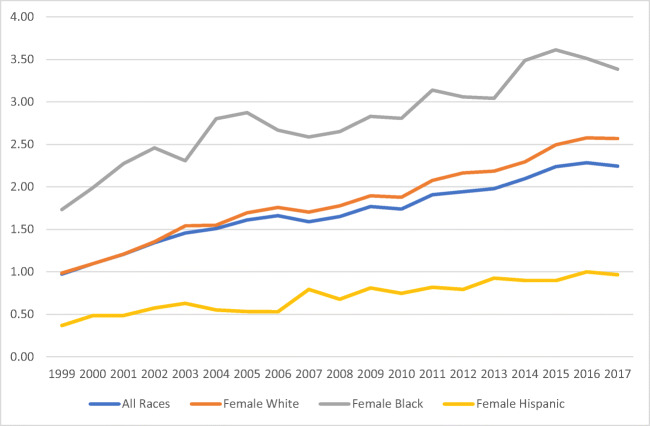
Female mortality rate* associated with obesity as an underlying cause of death in all three races/ethnic groups (White, Black, and Hispanic) in the USA from 1999 to 2017. *Rate per 100,000. Source: Mortality trends constructed from the US Mortality Database 1999–2017. Population obtained from the CDC WONDER database platform

Overall, the mortality rate trend in the period of study increased by 221.6% (0.74 to 2.38/1,000,000). The White population had an increased mortality rate of 183.7%, followed by Hispanics (165.0%) and Blacks (124.7%) respectively (Fig. 1). When mortality in the study period was separated by sex, males’ mortality rates were higher than females’ (Figs. 2 and 3), with the exception of Black females. Hispanics had the lowest mortality rates for both females and males.

In the USA, the mean age of death from any cause was 72.7, but if the underlying cause was obesity, this mean decreased to 56.5 years, resulting in a reduction of 16.2 years of life (Table 1b). It appears that when NCDs are associated with obesity as an underlying cause of death, the mean age of death from all NCDs drops from 74.9 to 57.3 years.

When comparing the mean age of death by race/ethnicity, we found that Hispanic individuals died 11 years earlier than White individuals and 0.9 years earlier than Black individuals, when obesity was an underlying cause (Table 1b).

When analyzing the age of death from all underlying causes by age group and race/ethnicity, we found that more than half of Black and Hispanic individuals died for any causes of death at younger ages (before age 70), while almost 70% of deaths among White individuals occurred after 70 years of age (Table 1c). When obesity was the underlying cause of death, more than 85% of Black and Hispanic individuals died before 70 years of age compared with 78% of White individuals. If obesity was the underlying cause of death associated to one or more NCDs, more than 40% of Hispanic and Black individuals died at ages younger than 70, compared to 28% of White individuals (Table 1c).

The highest mortality rates for obesity and associated conditions (per 1,000,000 inhabitants) for the 1999–2017 period were for cardiovascular disease, followed by diabetes, chronic respiratory disease, and cancer. Black individuals had the highest mortality rates for all four NCDs except cancer, while Hispanic individuals had the lowest rates for the four selected causes of death (Table 1d).

### Mortality Hazard Ratios for Obesity and Selected Associated Noncommunicable Chronic Diseases

The highest HRs for obesity and associated conditions for the 1999–2017 period were for diabetes (2.15), followed by cardiovascular disease (1.60), chronic respiratory disease (1.22), and cancer (0.05). Whites had the highest HR for diabetes, while Black individuals had the highest mortality HR for cardiovascular disease and chronic respiratory disease, and Hispanic individuals had the lowest HR for the selected causes of death except chronic respiratory disease. In all ethnic groups, the cancer mortality HR was less than one (Table 2a).

**Table 2:** Mortality hazard ratio for obesity as underlying cause and NCDs by race/ethnicity, USA 1999–2017

**2a. Mortality Hazard Ratio for Obesity and selected Chronic Non-Transmissible Communicable Disease**								
**Race/Ethnicity**		**All ethnic groups**	**White**		**Black**	**Hispanic**		
Cardiovascular Disease		1.6	1.57		1.81	1.21		
95% CI		1.58 - 1.62	1.55 - 1.60		1.75- 1.87	1.15 - 1.27		
Diabetes		2.15	2.46		1.32	1.25		
95% CI		2.11 - 2.18	2.41 - 2.51		1.27 - 1.38	1.18 - 1.33		
Cancer		0.05	0.05		0.03	0.03		
95% CI		0.04 - 0.05	0.05 - 0.06		0.03 - 0.04	0.02 - 0.04		
Chronic Respiratory Disease		1.22	1.23		1.62	1.41		
95% CI		1.20 - 1.25	1.20 - 1.26		1.55 - 1.69	1.31 - 1.52		
**2b. Mortality Hazard Ratio for Obesity and Cardiovascular Disease**								
**Age group**	**All Ethnic groups**		**White**		**Black**		**Hispanic**	
	**Male**	**Female**	**Male**	**Female**	**Male**	**Female**	**Male**	**Female**
0-14	5.99	3.63	5.83	3.42	8.53	3.5	5.57	2.15
95% CI	3.45- 10.38	1.83 - 7.20	2.62 - 12.99	1.08 - 10.81	2.67 - 24.93	1.01 - 12.11	1.70 - 18.29	0.54 - 8.60
15-29	11.22	5.5	10.79	5.75	16.71	4.58	8.17	4.37
95% CI	10.28 - 12.25	4.94 - 6.12	9.51 - 12.25	4.91 - 6.73	13.95 - 20.04	3.84 - 5.45	6.73 - 9.91	3.25 - 5.88
30-49	3.32	3.2	3.47	3.43	3.2	2.72	2.84	2.32
95% CI	3.21 - 3.44	3.08 - 3.33	3.33 - 3.62	3.27 - 3.60	2.96 - 3.45	2.53 - 2.93	2.57- 3.13	2.05 - 2.63
50-69	2.71	2.35	2.84	2.43	2.8	2.12	2.07	1.66
95% CI	2.63- 2.79	2.28 - 2.41	2.76 - 2.90	2.36 - 2.51	2.56 - 3.07	1.99 - 2.26	1.83- 2.35	1.48 - 1.85
70-84	2.1	1.56	2.85	1.53	2.68	1.69	1.73	1.49
95% CI	1.98 - 2.22	1.50 - 1.63	1.96 - 2.22	1.46 - 1.60	2.16 - 3.36	1.49 - 1.89	1.29- 2.32	1.23- 1.80
>85	1.68	1.41	1.63	1.37	3.19	1.53	1.21	1.54
95% CI	1.38 - 2.03	1.27- 1.56	1.33 - 2.00	1.22 - 1.53	1.24 - 8.23	1.13 - 2.07	0.50 - 2.87	1.01- 2.34
**2c. Mortality Hazard Ratio for Obesity and Diabetes**								
**Age group**	**All Ethnic groups**		**White**		**Black**		**Hispanic**	
	**Male**	**Female**	**Male**	**Female**	**Male**	**Female**	**Male**	**Female**
0-14	2.765	0.049	0.05	0.05	8.3	0.05	0.049	0.049
95% CI	0.38- 20.03	0.00 - 2.42e3	0.00 – 9.56e5	0.00- 6.06e8	1.04 - 62.25	0.00 – 1.02e6	0.00 - 8644	0.00 – 2.77e11
15-29	4.03	2.53	3.65	3.35	3.53	1.39	5.76	2.54
95% CI	3.37 - 4.81	2.07 - 3.07	2.75 - 4.85	2.52 - 4.43	2.61 - 4.79	1.00 - 1.93	3.97 - 8.36	1.44 - 4.48
30-49	1.86	2.16	2.29	2.52	1.56	1.63	1.66	1.47
95% CI	1.77 - 1.95	2.05- 2.27	1.91 - 2.16	2.36 - 2.69	1.41 - 1.73	1.46 - 1.79	1.45- 1.90	1.23 - 1.75
50-69	1.89	2.02	2.1	2.37	1.47	1.33	1.29	1.42
95% CI	1.84 - 1.95	1.96 - 2.08	2.03 - 2.17	2.29 - 2.46	1.34 - 1.61	1.24 - 1.49	1.12- 1.47	1.27 - 1.60
70-84	2.46	2.3	2.64	2.58	1.8	1.46	1.65	1.57
95% CI	2.32 - 2.61	2.20 - 2.41	2.48 - 2.82	2.44 - 2.71	1.45 - 2.22	1.29 - 1.65	1.23 - 2.20	1.30 - 1.90
>85	3.32	2.51	3.56	2.78	2.1	1.36	2.8	1.83
95% CI	2.71- 4.07	2.20 - 2.85	2.87 - 4.41	2.41 - 3.21	0.87 - 5.06	0.92 - 1.98	1.11 - 7.03	1.15 - 2.89
**2d. Mortality Hazard Ratio for Obesity and Cancer**								
**Age group**	**All Ethnic groups**		**White**		**Black**		**Hispanic**	
	**Male**	**Female**	**Male**	**Female**	**Male**	**Female**	**Male**	**Female**
0-14	0.04	0.04	0.04	0.04	0.04	0.04	0.04	0.04
95% CI	0.00 - 1.55	0.00 - 2.39	0.00 - 8.64	0.00 - 31.84	0.00 - 159.60	0.00 - 192.20	0.00 - 48.56	0.00 - 51.58
15-29	0.03	0.01	0.03	0.01	0.04	0.04	0.05	0.03
95% CI	0.01 - 0.08	0.00 - 0.04	0.01 - 0.14	0.002 - 0.09	0.01 - 0.30	0.01 - 0.15	0.01 - 0.28	0.00 - 0.23
30-49	0.02	0.01	0.02	0.01	0.01	0.02	0.03	0.01
95% CI	0.02 - 0.02	0.01 - 0.02	0.02 - 0.03	0.01 - 0.02	0.01 - 0.03	0.01 - 0.02	0.01 - 0.05	0.01 - 0.03
50-69	0.03	0.03	0.03	0.03	0.02	0.03	0.02	0.03
95% CI	0.02 - 0.03	0.02 - 0.03	0.023 - 0.03	0.02 - 0.03	0.02 - 0.03	0.03 - 0.04	0.0 - 0.04	0.02 - 0.04
70-84	0.08	0.08	0.08	0.08	0.1	0.07	0.08	0.04
95% CI	0.07 - 0.09	0.07 - 0.09	0.07 - 0.09	0.07 - 0.10	0.06 - 0.15	0.05 - 0.10	0.04- 0.17	0.02 - 0.09
>85	0.18	0.22	0.17	0.25	0.36	0.14	0.21	0.07
95% CI	0.12- 0.28	0.17- 0.30	0.11 - 0.28	0.18-0.34	0.11 - 1.17	0.05 - 0.38	0.03 - 1.57	0.01 - 0.53
**2e. Mortality Hazard Ratio for Obesity and Chronic Respiratory Disease**								
**Age group**	**All Ethnic groups**		**White**		**Black**		**Hispanic**	
	**Male**	**Female**	**Male**	**Female**	**Male**	**Female**	**Male**	**Female**
0-14	2.5	3.78	0.05	10.9	3.03	0.05	5.18	7.01
95% CI	0.78- 8.05	1.15 - 12.38	0.00 - 1.82e3	2.39 - 49.78	0.67 - 13.72	0.00 - 7314.27	0.66 - 40.73	0.87- 56.22
15-29	6.22	4.42	6.75	4.84	5.32	3.41	5.42	3.08
95% CI	5.16 - 7.49	3.57 - 5.47	5.10 - 8.93	3.51 - 6.69	3.91 - 7.22	2.47 - 4.71	3.24 - 9.08	1.37 - 6.96
30-49	2.3	2.61	3.47	3.43	2.32	2.81	3.39	3.7
95% CI	2.17 - 2.45	2.47 - 2.76	3.33 - 3.62	3.29 - 3.60	2.03 - 2.67	2.53 - 3.12	2.73 - 4.20	3.00 - 4.57
50-69	1.14	1.3	1.06	1.15	1.32	2.15	1.81	2.57
95% CI	1.10 - 1.18	1.25 - 1.34	1.02 - 1.10	1.11 - 1.20	1.17 - 1.49	1.99 - 2.36	1.51 - 2.17	2.21 - 2.98
70-84	1.52	1.51	1.46	1.43	1.61	2.46	2.55	2.57
95% CI	1.43- 1.61	1.44 - 1.59	1.37 - 1.56	1.36 - 1.51	1.27 - 2.05	2.11 - 2.77	1.89 - 3.44	2.07 - 3.19
>85	2.18	2.32	2.17	2.29	1.01	3.26	5	2.54
95% CI	1.78- 2.68	2.05 - 2.63	1.75 - 2.70	1.99 - 2.63	0.31 - 3.31	2.24 - 4.72	2.21 - 11.32	1.59 - 4.06

### Mortality Hazard Ratio for Obesity and Associated Cardiovascular Disease

The overall obesity mortality HRs by sex, age group, and race/ethnic origin for the associated cardiovascular disease condition were higher among males than females, with the exception of Hispanic females in the over 85 years of age group, where the mortality HR was higher than that of males in the same age group. The 0–14 and 15–29 age group had the highest HRs for males in all race/ethnic groups. In this study, obesity and cardiovascular disease mortality HRs decreased with age (Table 2b).

### Mortality Hazard Ratio for Obesity and Associated Diabetes

For all three races/ethnic groups, mortality HRs for obesity and the associated diabetes were higher among males than female in all ages group, except for the 30–49 and 50–69 age groups. HRs were higher among males between the ages of 15 and 29 and over 85 years of age. Hispanic and Black males had almost double the mortality HRs of Hispanic and Black women in the 15–29 age group, while White and Hispanic females had higher HRs in the 30–49 age group and 50–69 age group. In general, the mortality HR increased with age (Table 2c).

### Mortality Hazard Ratio for Obesity and Associated Cancer

In all age groups, HRs were below one for obesity mortality and cancer (Table 2d). HR increases with age after age 50.

### Mortality Hazard Ratio for Obesity and Associated Chronic Respiratory Disease

The mortality HRs for obesity and associated chronic respiratory disease in all races and Hispanic origin decreases between the 15–29 and 50–69 age groups, and then start to increase from the age of 70 and above. The HRs were higher in females than in males in the 0–14 age group, except for Black females; in the 15–29 age group, the mortality HRs were higher among males than females in all races and Hispanic origin ethnic groups. Male Hispanics had higher mortality HRs than White and Black individuals in all age groups, except for males in the 15–29 to 30–49 age groups (Table 2e); Female Hispanics had higher mortality HRs than White and Black individuals between the ages of 30 and 84.

## Discussion

In this study, although the relative percentage seems to be small, 0.2% accounts for 99.388 cases, which was large enough for inferential analyses by Cox regression. With this type of analysis, rather than focusing on pooled proportions, accounts for comparisons of hazards, and instantaneous risk rates of time to death, we found that people who have obesity as an underlying cause of death die, on average, 16.2 years younger than those without the condition [30]. We found that obese Hispanic individuals die on average 6.8 years earlier than White individuals and 0.9 years earlier than Black individuals. Several studies have documented that mild and severe obesity are associated with the loss of one in ten and one in four potential disease-free years during adulthood, respectively [12, 30, 31]. Our 19 years of analyzed data showed a steady increase in the obesity mortality trend among the three main races/ethnic groups, while other studies document that overall mortality among obese persons is declining over time [31, 32]. More research is needed on obesity mortality when it is part of multiple conditions in death certificates.

We found that even though Hispanic individuals in the USA live in disadvantaged conditions, with a higher prevalence of diabetes and cardiovascular diseases, they have the lowest mortality HRs for obesity as the underlying cause of death with cardiovascular diseases and diabetes [32–38]. Many studies about the Hispanic paradox have tried to explain why this group has lower mortality, but none has fully explained the reasons for these ambivalences. Although White individuals had a higher prevalence of cancer and chronic respiratory disease [35], in this study, we found that Hispanic individuals between the ages of 30 and 84 had higher mortality HRs for chronic respiratory disease. We found that the HRs for obesity and cancer, in most cases, were below one in the three race/ethnic groups, suggesting that it is possible that the role of overall obesity is overlooked in cancer mortality, possibly because of the weight loss resulting from cancer [5]. More research is needed to understand the reason for the low HR, as it is known that there are some forms of cancer that have an increased risk if obesity is observed (breast, endometrial, and pancreatic cancer) [39].

As mentioned before, although the use of ICD codes has a positive prediction value of more than 90%, the present study has the limitation that we only used mortality datasets without further information about BMI, obesity class, and other risk factors such as physical activity or smoking, as the existing data in the mortality datasets were either incomplete or unavailable; therefore, their effect and relationship with NCDs cannot be evaluated [40]. While we could not establish how these risk factors impact obesity mortality, it has been well-documented that smoking, unhealthy eating, and physical inactivity play an important role in higher BMI levels, and as such are contributing factors for adverse health outcome [41]. In this study, deaths from cardiovascular conditions and cancer were not disaggregated into specific types, and mortality hazard ratios were estimated only for obesity and the selected major chronic conditions.

A strength of this study, however, is the analysis of a database of almost 48 million death and the three-principal race/ethnic groups in the USA (White, Black, and Hispanic).

## Conclusions

Our findings of higher HRs in the younger age groups should be considered to develop interventions to control and prevent obesity and/or delay the development of complications that may lead to premature death, as mentioned by other authors and confirmed in this study [3, 5].

Although there is an increasing trend of obesity mortality in the USA [4], it is difficult to say if the trend is real or if it has occurred because there is more awareness about this problem among health professionals who use the E66 code more frequently. Further research is needed to establish the cause of this increase in obesity mortality.

As shown in Table 2b, 2c, and 2e, HRs were higher in all three races/ethnic groups (males have HRs higher than females) for cardiovascular disease, diabetes, and chronic respiratory disease in the 15–29 year age group than in any other older age group; it is therefore necessary that the public health programs increase their efforts to promote weight management among all obese individuals, with an emphasis on younger groups.

We agree with the conclusion of The Lancet Commission on Obesity, which states that there is not an obvious solution for obesity and its complication [2]; consequently, continued efforts are needed to address the obesity and its corresponding public health and policy concerns in order to prevent the increase of disabilities associated with obesity over time [42].

Finally, in the context of the COVID-19 pandemic, evidence suggests that obesity is an important risk factor not just for noncommunicable diseases but also for communicable ones as well. Obesity has also been reported to play an important role in contributing to higher mortality rates among Hispanic and Black people, where the socioeconomic factor is likely to have some influence on timely access to health services [43]. An effective public health response for addressing obesity is consequently more urgent than ever.

## Data Availability

Database is available upon request.
